# 

**Published:** 1895-10

**Authors:** 


### PERSONAL.

Dr. D. LeMay has just returned to his post from a trip to California for the inspection and purchase of cavalry horses.

Dr. James A. Waugh is out in a timely article in the Pittsburg *Post*, reviewing the subject of rabies and hydrophobia, with a view to allaying the excitement and undue anxiety now prevailing in that community.

State Veterinarian Turner, of Missouri, will shortly issue a bulletin relative to the extent and character of prevalent diseases among live stock in that State the past year, with many suggestions to the owners as to the early recognition and management of these outbreaks.

Dr. H. F. James has been appointed veterinarian to the St. Louis (Mo.) City Board of Health.

Dr. James A. Waugh will prepare a paper for the next annual meeting of the Pennsylvania State Veterinary Medical Association, entitled "The American Veterinary Trade."

Veterinarian W. B. Wallace, of Marion, Ind., will again assemble his professional colleagues at Muncie in December next to partake of a specially prepared dinner of horseflesh.

Dr. Ulysses G. Houck succeeds Dr. Henry Marshall as house surgeon to the Veterinary Department of the University of Pennsylvania.

Prof. H. J. Detmers, of Columbus, O., has been visiting Philadelphia for the purpose of negotiating with one of the medical book publishers there, looking to the issue of his work on pathology.

Among the leading Knights Templar at the annual conclave in Boston were to be found the well-known veterinarian, Dr. J. E. Cloud, of Richmond, President of the Indiana Veterinary Association of Graduates.

Dr. Henry Marshall, formerly house surgeon of the Veterinary Department, University of Pennsylvania, has accepted an assistantship with Prof. Leonard Pearson, of Philadelphia.

Drs. William Sheppard, Edward Loomes, and Thomas G. Sherwood will officiate as veterinary inspectors at the coming horse show in New York, November 11 to 16, 1895.

